# Mechanical properties of murine hippocampal subregions investigated by atomic force microscopy and in vivo magnetic resonance elastography

**DOI:** 10.1038/s41598-022-21105-7

**Published:** 2022-10-06

**Authors:** Anna S. Morr, Marcin Nowicki, Gergely Bertalan, Rafaela Vieira Silva, Carmen Infante Duarte, Stefan Paul Koch, Philipp Boehm-Sturm, Ute Krügel, Jürgen Braun, Barbara Steiner, Josef A. Käs, Thomas Fuhs, Ingolf Sack

**Affiliations:** 1 Department of Radiology, Charité-Universitätsmedizin Berlin, Corporate Member of Freie Universität Berlin and Humboldt-Universität zu Berlin, Charitéplatz 1, 10117 Berlin, Germany; 2 Institute of Anatomy, University of Leipzig, Leipzig, Germany; 3 Experimental and Clinical Research Center, Max Delbrück Center for Molecular Medicine in the Helmholtz Association (MDC) and Charité-Universitätsmedizin Berlin, Corporate Member of Freie Universität Berlin and Humboldt-Universität zu Berlin, Berlin, Germany; 4 Einstein Center for Neurosciences Berlin, Charité-Universitätsmedizin Berlin, Corporate Member of Freie Universität Berlin and Humboldt-Universität zu Berlin, Berlin, Germany; 5 Department of Experimental Neurology and Center for Stroke Research, Charité-Universitätsmedizin Berlin, Corporate Member of Freie Universität Berlin and Humboldt-Universität zu Berlin, Berlin, Germany; 6 NeuroCure Cluster of Excellence and Charité Core Facility 7T Experimental MRIs, Charité-Universitätsmedizin Berlin, Corporate Member of Freie Universität Berlin and Humboldt-Universität zu Berlin, Berlin, Germany; 7 Rudolf Boehm Institute of Pharmacology and Toxicology, University of Leipzig, Leipzig, Germany; 8 Institute of Medical Informatics, Charité-Universitätsmedizin Berlin, Corporate Member of Freie Universität Berlin and Humboldt-Universität zu Berlin, Berlin, Germany; 9 Clinic for Neurology and Experimental Neurology, Charité-Universitätsmedizin Berlin, Corporate Member of Freie Universität Berlin and Humboldt-Universität zu Berlin, Berlin, Germany; 10 Section of Soft Matter Physics, Peter Debye Institute for Soft Matter Physics, Faculty of Physics and Geosciences, University of Leipzig, Leipzig, Germany

**Keywords:** Biophysics, Neuroscience, Physics

## Abstract

The hippocampus is a very heterogeneous brain structure with different mechanical properties reflecting its functional variety. In particular, adult neurogenesis in rodent hippocampus has been associated with specific viscoelastic properties in vivo and ex vivo. Here, we study the microscopic mechanical properties of hippocampal subregions using ex vivo atomic force microscopy (AFM) in correlation with the expression of GFP in presence of the nestin promoter, providing a marker of neurogenic activity. We further use magnetic resonance elastography (MRE) to investigate whether in vivo mechanical properties reveal similar spatial patterns, however, on a much coarser scale. AFM showed that tissue stiffness increases with increasing distance from the subgranular zone (p = 0.0069), and that stiffness is 39% lower in GFP than non-GFP regions (p = 0.0004). Consistently, MRE showed that dentate gyrus is, on average, softer than Ammon´s horn (shear wave speed = 3.2 ± 0.2 m/s versus 4.4 ± 0.3 m/s, p = 0.01) with another 3.4% decrease towards the subgranular zone (p = 0.0001). The marked reduction in stiffness measured by AFM in areas of high neurogenic activity is consistent with softer MRE values, indicating the sensitivity of macroscopic mechanical properties in vivo to micromechanical structures as formed by the neurogenic niche of the hippocampus.

## Introduction

Neural homeostasis, repair, and circuit function require that new neurons be formed throughout the lifespan^1,2^. In rodents, adult neurogenesis takes place in different brain regions such as the hippocampus, specifically in the subgranular zone (SGZ) of the dentate gyrus (DG). New neurons are formed from progenitor cells, whose proliferation, morphogenesis, and differentiation are tightly controlled by chemical and physical cues^3^. While chemical cues for neural stem cell differentiation, such as epidermal or fibroblast growth factors^4,5^, have been extensively studied, the importance of physical control of neural development remains understudied^6^. For instance, it is known that substrate stiffness influences proliferation and differentiation of neural progenitor cells, axonal growth, branching, and maturation^7–15^; however, little is known about the viscoelastic properties of the murine SGZ in vivo.

While ex vivo methods such as atomic force microscopy (AFM) can measure the mechanical properties of hippocampus subzones with a spatial resolution on the order of a few microns^16^, in vivo methods such as magnetic resonance elastography (MRE) rely on coarser scales of submillimeters in the murine brain^17^ and millimeters in humans^18^. MRE uses externally induced shear waves for noninvasively probing tissue viscoelasticity as a quantitative diagnostic marker^19,20^. Although MRE lacks microscopic spatial resolution, the shear modulus of soft biological tissues is sensitive to multiscale mechanical structures from microscopic cellular dimension to macroscopic length scale^18,21–24^. Thus, macroscopic viscoelastic parameters of brain tissues are sensitive to a concert of interactions among neural cells, extracellular matrix (ECM), and vessels^25–28^. In vivo MRE has been shown to be sensitive enough to detect subtle changes in neurogenesis in the murine hippocampus induced by a Parkinson's disease model^29,30^. However, the association between MRE parameters and physiologic, adult neurogenesis in hippocampal subregions such as the neurogenic niche, the SGZ, has never been investigated. Furthermore, AFM findings in a similar region as studied before by in vivo MRE have never been correlated with neurogenic function.

Pairing AFM with MRE, on the one hand, allows us to correlate stiffness of micro tissue with the level of expression of green florescence protein (GFP)-labelled nestin, as a marker of neural progenitor^31^ cells, and, thus, to directly test the mechanical properties of the neurogenic niche ex vivo. On the other hand, the study of viscoelastic properties by in vivo MRE takes into account vital processes such as blood flow^32^, neural activity^33^, and metabolism^32^, which are absent when tissues are studied ex vivo*.* Therefore, here we compare AFM with in vivo MRE for the first time to test whether the coarse resolution of MRE reveals a similar mechanical signature as detected by AFM at the microscopic level and in correlation with neurogenic activity. This study aims at contributing to our understanding of the micromechanical parameters that critically shape the biophysical environment of neuronal homeostasis and repair and could be exploited for neuronal regenerative medicine in the future.

## Material and methods

### In vivo MRE and MRI

All animal experiments were carried out in accordance with Directive 2010/63/EU of the European Parliament and of the Council of 22 September 2010 and national and institutional guidelines for the care and use of laboratory animals and were approved by the local Animal Ethics Committee (State Office for Health and Social Affairs Berlin, Germany and State Ministry for Social Affairs and Consumer Protection, Leipzig, Germany). Methods are provided in accordance with ARRIVE guidelines. A total of 10 C57BL/6J mice (male, 9–10 weeks old) were investigated by MRE.

MRE and magnetic resonance imaging (MRI) were performed on a 7-Tesla small animal MRI scanner (Bruker BioSpec, Ettlingen, Germany). During the scans, mice were anesthetized with 1.5–2% isoflurane in 30% O_2_ and 70% N_2_O. The gas was administered through an anesthesia mask, and respiration was continuously monitored using a pressure-sensitive pad placed on the thorax (Small Animal Instruments Inc., Stony Brook, NY, USA). Body temperature was kept constant through warming pads integrated into the animal holder and monitored using a rectal probe. A 20-mm (mm) diameter 1H-RF transmit/receive quadrature volume coil (RAPID Biomedical, Würzburg, Germany) was used for MRI and MRE.

**Figure 1:**
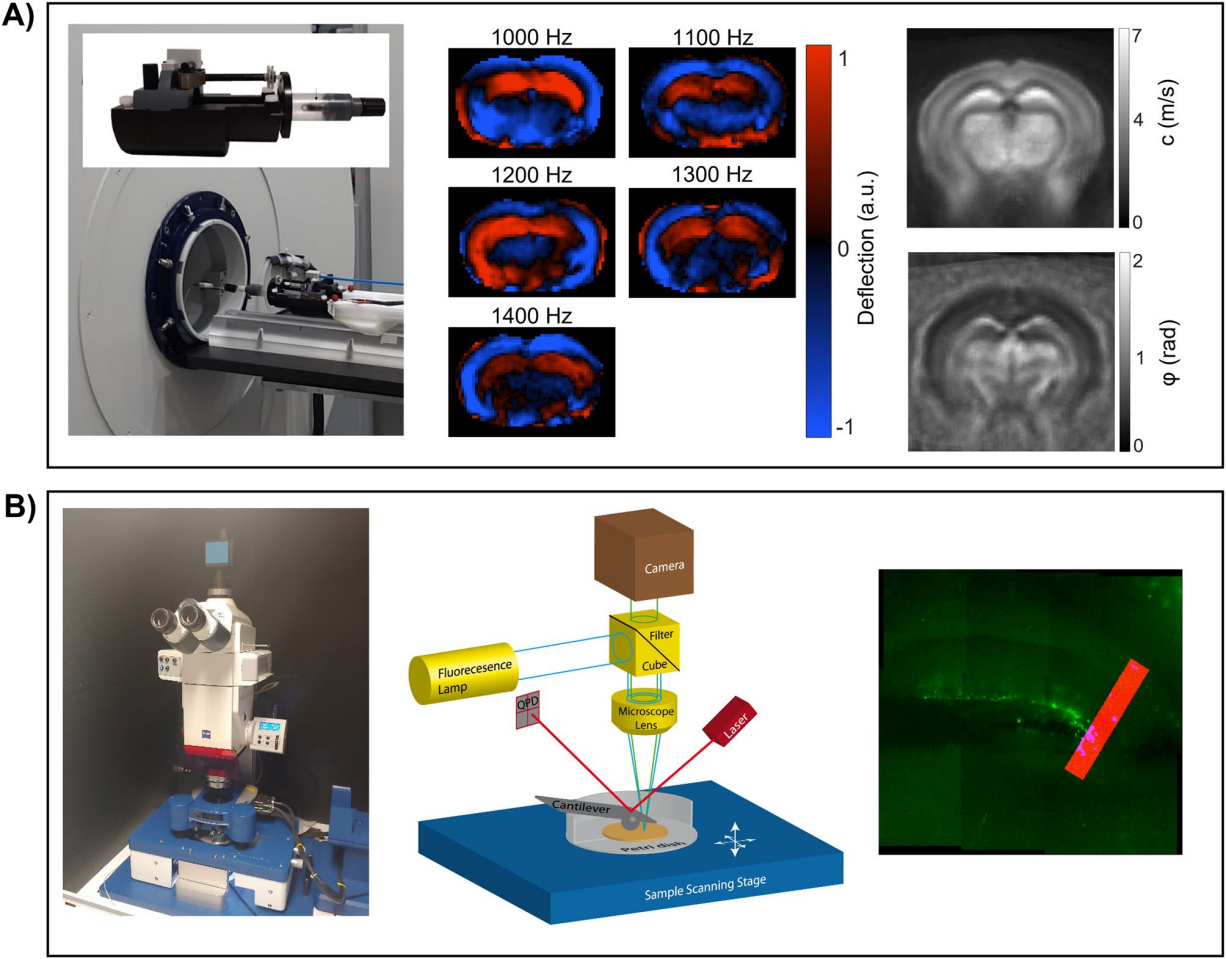
MRE and AFM setup. (**A**) On the left, the 7-Tesla animal scanner is shown along with customized animal holder, comprising a nonmagnetic piezoceramic actuator for wave generation, transducer rot (for wave transmission into the animal`s skull), and an anesthesia mask. In the middle, wave images showing shear wave propagation in the mouse brain at the 5 vibration frequencies (after k-MDEV inversion, 1 slice, 1 component, and no directional filters applied). On the right, a representative averaged MRE c-map and φ-map are shown. (**B**) On the left, the atomic force microscopy setup, in the middle a schematic drawing of the AFM, illustrating the main components of an AFM, and on the right, a representative image of a brain slice with a measurement profile are shown.

MRE was performed based on five consecutively induced and encoded vibration frequencies of 1000, 1100, 1200, 1300, and 1400 Hz. Vibrations were generated by a piezoceramic actuator and transmitted into the skull via a transducer rod as shown in Fig. 1A. A total of 7 coronal slices (in the Bregma areas − 2.84 mm (mm) to 0.23 mm) with 0.8 mm slice thickness were acquired using a single-shot echo-planar imaging sequence as described in detail in^17^. Further imaging parameters were: 0.18 mm × 0.18 mm in-plane resolution, echo time (TE) = 53 ms (ms), repetition time (TR) = 4000 ms, 16.2 × 10.8 mm^2^ field of view (FOV), total scan time (TA) of 9 min (min). Tomoelastography postprocessing^34^ was performed to derive shear wave speed (c in m/s) as a surrogate marker of stiffness, based on multifrequency wave-number analysis. To recover the phase angle of the complex shear modulus (φ in rad, also termed loss angle), Laplace-based direct multifrequency inversion was used^20^. To match AFM measurement, parameters c and φ (Fig. 1A right) were analyzed in bregma area − 2.4945 mm to − 2.0745 mm. Furthermore, c and φ were converted to shear stiffness (magnitude of the complex shear modulus |G*| in Pa) by

1 $$\left| {G^{*} } \right| = \frac{{c^{2} \rho \left( {1 + \cos \varphi } \right)}}{2},$$

assuming material density ρ = 1000 kg/m^3^. For comparison with AFM-measured Young’s modulus *E*, we tabulated in vivo Young’s modulus *E*_MRE_ = 3·|G*|.

For anatomical orientation, 13 coronal slices covering the Bregma areas − 2.84 to 0.23 mm were acquired with a standard T2-weighted 2D rapid acquisition with relaxation enhancement (RARE) MRI sequence. Imaging parameters were: 0.8 mm slice thickness, 0.078 mm × 0.078 mm in-plane resolution, TE = 33 ms, TR = 2500 ms, 20 mm × 20 mm FOV, 265 × 265 matrix size, RARE factor = 2, number of averages = 2, bandwidth = 34,722 Hz, and TA of 2:45 min.

### Image registration and generation of masks

MRE parameter maps of c and φ and the corresponding MRE magnitude images were registered to the Allen mouse brain atlas (^35^ Allen Institute for Brain Science, United States Allen Mouse Brain Atlas (2017) available from: https://mouse.brain-map.org) using ANTx2, a customized MATLAB toolbox (latest version available from https://github.com/ChariteExpMri/antx2), as described elsewhere^26^. In short, MRE parameter maps and magnitude images were 3D-coregistered and registered to each animal’s T2-weighted MR image in a 2D slice-wise fashion using affine and non-linear b-spline transformations. In a next step, affine and non-linear b-spline registrations were applied to bring the animal’s T2-weighted MR image to the Allen mouse brain atlas space (standard space) using ELASTIX (http://elastix.isi.uu.nl/)^36^. Finally, the MRE parameter maps and magnitude images were transformed to standard space by applying the registration parameters from the previous step.

Standard space masks of the hippocampus and DG were generated in ANTx2. To obtain a mask of Ammon´s horn, the DG mask was cut out of the hippocampus mask using MATLAB R2019b (MathWorks Inc., Natick, MA, USA). To evaluate the subzones of the DG, the masks were 2D-eroded by one, two, and three pixels from the outer DG boundaries towards the SGZ in a slice-wise fashion using the imerode function of MATLAB R2019b (MathWorks Inc., Natick, MA, USA). Standardized DG and AH masks and eroded DG masks were overlaid on MRE parameter maps in the Bregma area − 2.4945 to − 2.0745 mm.

### Atomic force microscopy (AFM)

Six Tg(Nes-EGFP)33Enik/J mice (male, 7–9 weeks old), expressing the green fluorescence protein (GFP) under the nestin promoter, were euthanized with an isoflurane (CP-Pharma, Burgdorf, Germany) overdose and transcardially perfused with ice cold phosphate-buffered saline (PBS). Brains were removed and placed in artificial cerebral spinal fluid (aCSF, solution I: 124 millimols (mM) sodium chloride [NaCl], 1.25 mM sodium dihydrogen phosphate [NaH_2_PO_4_], 10.0 mM glucose, 1.8 mM magnesium sulphate [MgSO_4_)], 1.60 mM calcium chloride [CaCl_2_], and 3.00 mM potassium chloride [KCl]; solution II: 26.0 mM sodium bicarbonate [NaHCO_3_]. Solutions I and II were mixed immediately before use). Coronal brain slices of 350 μm thickness were cut, using a vibratome (Leica VT 1200, Leica Biosystems, Wetzlar, Germany) in the Bregma area − 2.4945 to − 2.0745 mm and placed on ice in a 24-well plate containing aCSF until further measurement. Next, indentation measurements were obtained by AFM (Fig. 1B) to determine the elastic properties of the hippocampus (Nanowizard4 with 300 µm hybrid stage, JPK, Berlin, Germany). Commercially available cantilevers (0.2 N/m, CONT, Nanoworld, Neuchatel, Swiss) were modified by gluing a small polystyrene bead (~ 6 μm diameter) onto the tip. During indentation measurement, the tissue was immobilized on a glass slide with surgical glue (Histoacryl, Braun, Melsungen, Germany) and placed in aCSF supplemented with synthetic air with 5% CO_2_. For each slice, a profile of 10–15 adjacent maps of 100 × 100 µm with a pixel resolution of 5–10 µm (100–225 pixels) was laid over the DG, comprising the SGZ. The force curves were recorded at 20 µm/s z-speed with the setpoint of 2 nN corresponding to around 2 µm indentation. AFM force–displacement curves were transformed to the local Young’s modulus using the Hertz model (*F* = force, *E* = Youngs modulus, *R* = tip radius, *d* = indentation depth):

2 $$F = \frac{4}{3}E \cdot R^{\frac{1}{2}} \cdot d^{\frac{3}{2}}$$

Supplementary Fig. 1 shows a fit of the Hertz model to experimental data and a series of experimental force-indentation curves. Total measurement time was below 2 h per slice. Fluorescence, visualizing the SGZ, was used for anatomical orientation (Fig. 1B). Young´s modulus (E in Pa) and local fluorescent intensity were acquired for each measurement point (Fig. 1B). For comparison based on local GFP expression, a threshold of relative fluorescent intensity was applied to automatically select all pixels within the AFM map which corresponded to the full width at half maximum of peak intensity within the GFP images. Elasticity values within such areas of high fluorescent signal were compared with values of the other areas which were considered of low fluorescent signal intensity. For position-based analysis, the map with the highest fluorescent intensity was selected as the central map, and averages were calculated for each map in the profile. Analysis was performed using Data Processing Software (JPK BioAFM—Bruker Nano GmbH, Berlin, Germany) and MATLAB (MathWorks Inc., Natick, MA, USA). A total of 15 measurements were performed.

### Immunohistochemistry and microscopy

One Tg(Nes-EGFP)33Enik/J mouse was deeply anesthetized with ketamine/xylazine at a dose of 300 mg/kg body weight ketamine hydrochloride (Ketamine Insera, Insera Arzneimittel, Freiburg, Germany) and 30 mg/kg body weight xylazine hydrochloride (CP-Pharma, Burgdorf, Germany) administered by intraperitoneal injection. Next, the mouse was transcardially perfused with 1 M (moles per liter) PBS and 4% paraformaldehyde (PFA), and the brain was extracted. The tissue was post-fixated in PFA overnight at 4 °C and placed in 30% sucrose for 72 h. To cut the tissue in 40 µm thick slices using a cryostat (Leica CM 1850 UV, Wetzlar, Germany), brains were frozen in 2-methylbutane cooled with liquid nitrogen. The brain slices were stored in cryoprotectant solution (25% glycerol, 25% ethylene glycol, and 50% 0.1 M PBS) at 4 °C until further use.

Immunohistochemistry was performed to visualize nestin-expressing cells in the hippocampus. Free-floating brain sections were washed with PBS and placed in 10% donkey serum-enriched PBS (PBS^+^) for 30 min at room temperature (RT). After blocking, sections were incubated overnight at RT with the primary antibody, anti-rabbit-GFP (1:250, Abcam, Cambridge United Kingdom) in PBS^+^. On the next day, sections were washed in PBS, incubated in PBS^+^ for 25 min at RT and subsequently incubated with the secondary antibody, anti-rabbit Alexa 488 (1:1000, Thermo Fischer Scientific, Waltham, MA, USA) for 4 h at RT. After washing, sections were incubated with PBS-diluted fluorochrome 4´6-diamidino-2-phenylindole (DAPI, 1:10,000, Thermo Fischer Scientific, Waltham, MA, USA) for 7 min to visualize cell nuclei. Tissue sections were mounted on microscope slides using ProTags PARAmount (Quartett GMBH, Berlin, Germany). Imaging was performed on a Keyence BZ-X800 microscope (Keyence, Osakaa, Japan). Images were postprocessed using BZ-X800 Analyzer software (Keyence, Osakaa, Japan).

### Statistics

Statistical analysis was performed using GraphPad Prism 9.0 (GraphPad software, San Diego, CA USA). The D'Agostino & Pearson test was used to test normality. A paired t-test was used to compare in vivo AH and DG viscoelastic differences and ex vivo elastic differences of low and high fluorescence signal. Repeated measures one-way ANOVA with multiple comparisons (Tukey's multiple comparisons test) was performed to compare differences in the dentate gyrus subzone in vivo while ex vivo analysis was perfomed using a mixed model. Values are presented as mean ± standard deviation (SD). Statistical significance was defined as p < 0.05.

## Results

**Figure 2:**
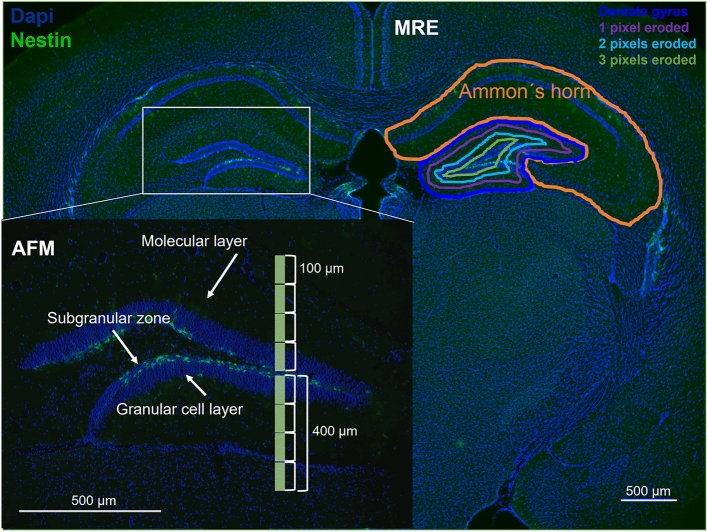
MRE masks and AFM measurement profiles. The MRE Ammon´s horn mask, dentate gyrus mask (DG), and DG mask eroded by 1, 2, and 3 pixels are schematically overlaid onto a histological image (magnification × 10). At the bottom, a schematic AFM measurement profile (in green) on the DG (magnification × 20). blue—DAPI (cell nuclei), green—nestin.

Figure 2 shows examples of MRE masks along with a schematic AFM profile superimposed on a histological image of the mouse brain stained for DAPI (cell nuclei) and nestin. The SGZ in the DG is clearly identified by the expression of green fluorescence nestin in neuronal stem cells and was covered by both, in vivo MRE and ex vivo AFM measurements.

### In vivo MRE is sensitive to micromechanical properties

**Figure 3:**
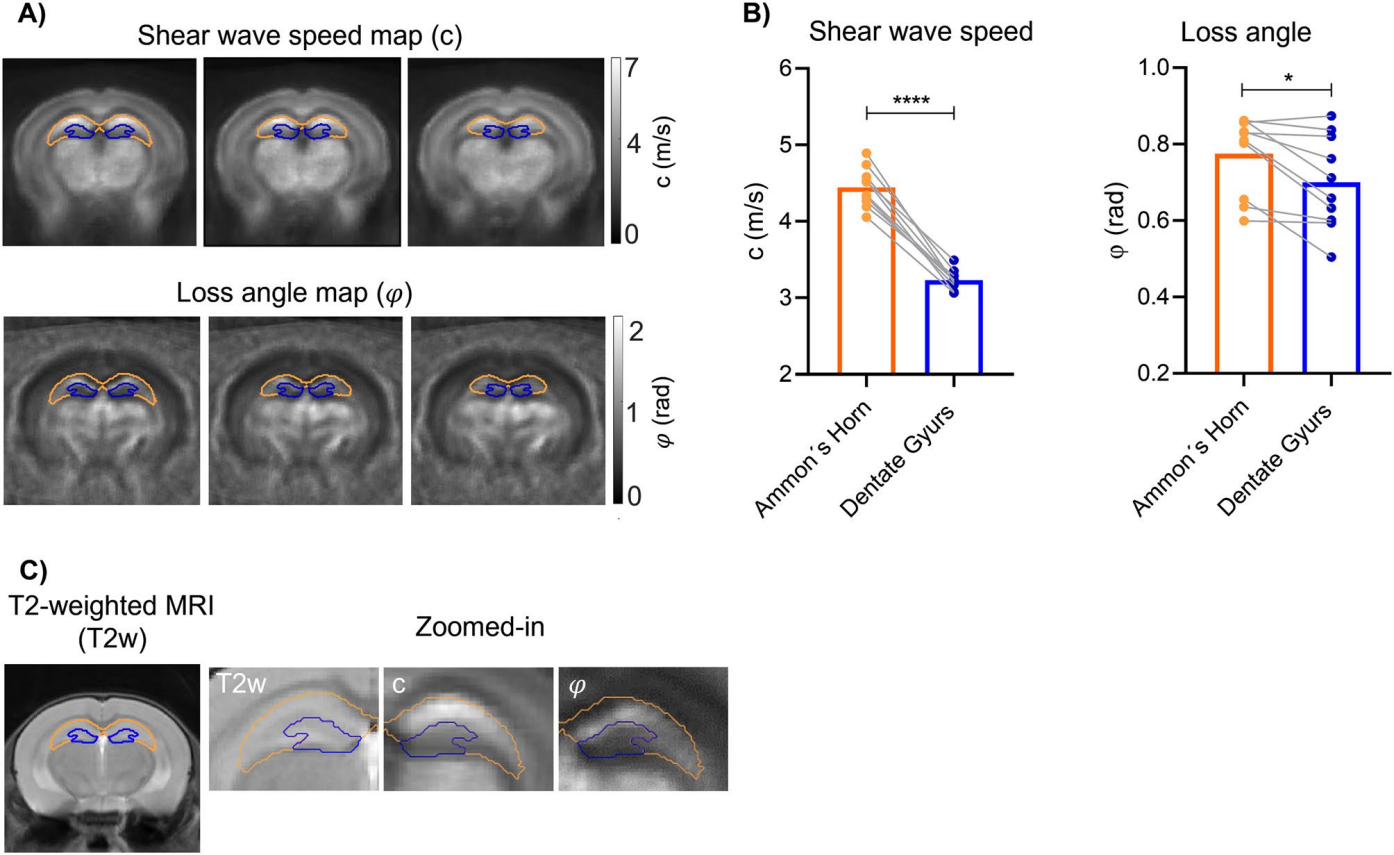
Regional in vivo MRE results. (**A**) Representative group averaged c- and φ-maps with Ammon´s horn (orange) and dentate gyrus (blue) masks. Image slices with distances of 210 µm correspond to the Bregma areas − 2.49, − 2.28 and − 2.07. (**B**) Group values of c and φ for Ammon´s horn and dentate gyrus (n = 10, *p = 0.0102, ****p < 0.0001). (**C**) T2-weighted MRI for anatomical orientation with magnifications of parameter maps and masks in the hippocampal region.

Figure 3 shows high resolution maps of c and φ obtained by averaging individual maps after image registration to the standard atlas of the mouse brain. The DG and AH are apparent as distinct regions with lower shear wave speed c (DG = 3.2 ± 0.2 m/s *vs.* AH = 4.4 ± 0.3 m/s, p < 0.0001, Fig. 3A) and loss angle φ (DG 0.70 ± 0.13 rad *vs*. AH 0.77 ± 0.11 rad, p = 0.01, Fig. 3B) in DG than in AH.

**Figure 4:**
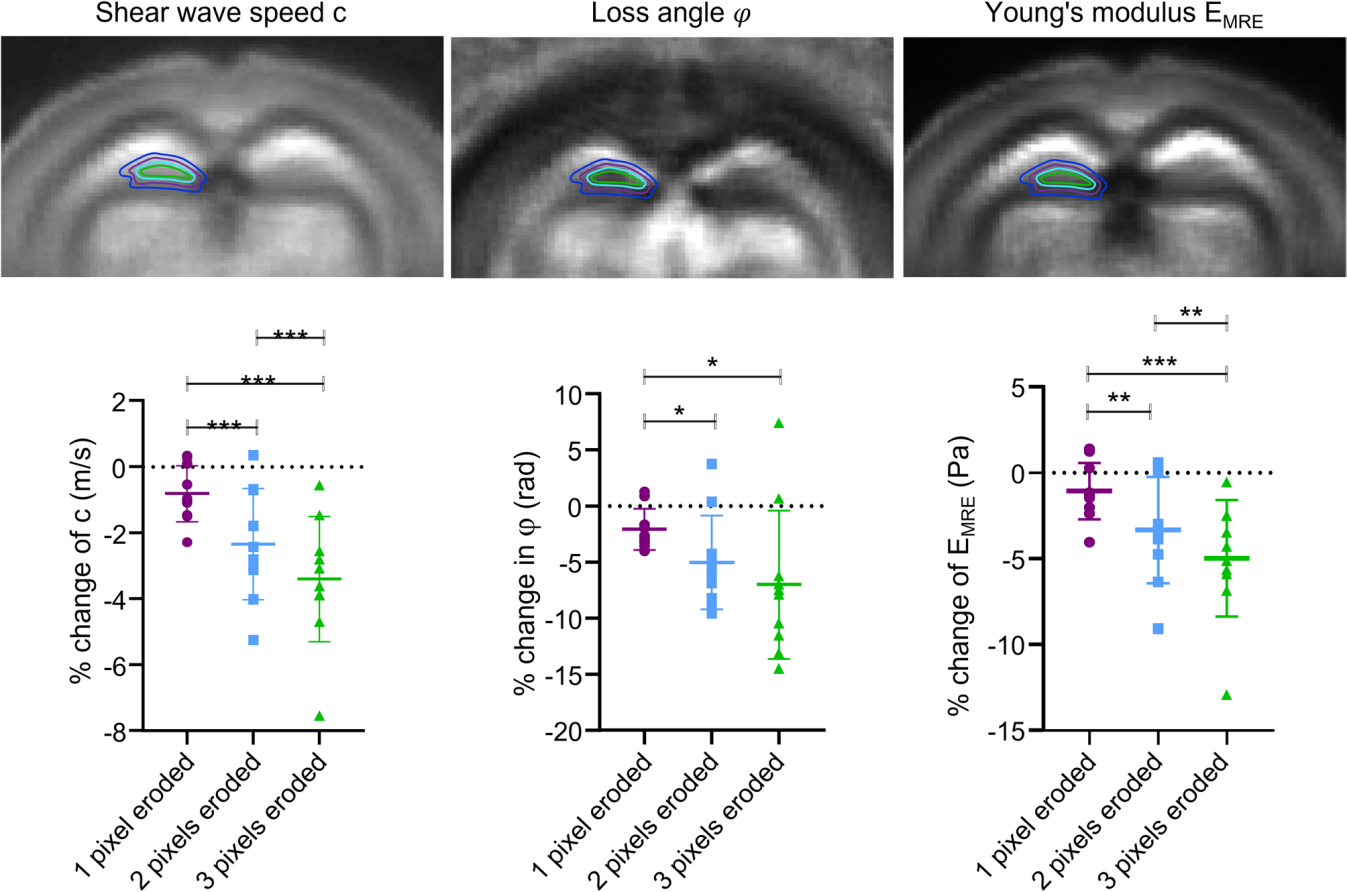
Eroding the dentate gyrus (DG) mask by 1, 2, and 3 pixels leads to significant decrease in viscoelastic parameters. Top, schematic mask of original DG and 1, 2, and 3 pixels eroded overlaid on averaged c-, φ- and EMRE-maps. Bottom, from left to right, percentage change in c (m/s) showing a significant decrease in c (m/s) when the DG mask is 1, 2 and 3 pixels (pixels) eroded (1 pixel eroded vs. 2 pixels eroded ***p = 0.0001, 1 pixel eroded vs. 3 pixels eroded ***p = 0.0001, 2 pixels eroded vs. 3 pixels eroded ***p = 0.0005); in the middle, percentage change in φ (rad) showing a significant decrease in φ (rad) when the DG mask is 2 and 3 pixels eroded (1 pixel eroded vs. 2 pixels eroded *p = 0.0108, 1 pixel eroded vs. 3 pixels eroded *p = 0.0270, 2 pixels eroded vs. 3 pixels eroded p = 0.0784); on the right, percentage change in EMRE (Pa) showing a significant decrease in E (Pa) when the DG mask is 1, 2 and 3 pixels eroded (1 pixel eroded vs. 2 pixels eroded **p = 0.0029, 1 pixel eroded vs. 3 pixels eroded ***p = 0.0004, 2 pixels eroded vs. 3 pixels eroded **p = 0.0046), n = 10.

Furthermore, Fig. 4 shows that eroding DG masks by 1, 2 and 3 pixels towards SGZ significantly reduces c, φ, and derived Young’s modulus *E*_MRE_. Table 1 summarizes absolute values. Relative changes are − 0.8 ± 0.9%, − 2.1 ± 1.9%, and − 1.1 ± 1.7% (one-pixel erosion), − 2.3 ± 1.7%, − 5.0 ± 4.2%, and − 3.3 ± 3.1% (two-pixel erosion), and − 3.4 ± 1.9%, − 6.9 ± 6.7% and − 5.0 ± 3.4% (three-pixel erosion) for c, φ, and derived Young’s modulus *E*_MRE_, respectively (significances are shown in Fig. 4).

**Table 1:** Mean and standard deviation (SD) for c (m/s), φ (rad) and Young’s modulus (kPa or Pa).

MRE				AFM	
	c (m/s)	φ (rad)	E_MRE_ (kPa)		E (Pa)
Mask	Mean ± SD	Mean ± SD	Mean ± SD	Distance (µm)	Mean ± SD
Ammon’s horn	4.4 ± 0.3	0.77 ± 0.11	50.8 ± 6.1	0	84 ± 40
Dentate Gyrus	3.2 ± 0.2	0.70 ± 0.13	27.7 ± 3.1	100	145 ± 82
1 pixel eroded	3.2 ± 0.2	0.68 ± 0.12	27.4 ± 3.2	200	177 ± 68
2 pixels eroded	3.2 ± 0.2	0.66 ± 0.12	26.8 ± 3.3	300	158 ± 80
3 pixels eroded	3.1 ± 0.2	0.65 ± 0.12	26.3 ± 3.3	400	142 ± 63

On the left, mean and SD of c (m/s) and φ (rad) and Young’s modulus (E_MRE_ = 3 · |G*|) of the Ammon’s horn and DG masks and the DG masks reduced by 1 to 3 pixels from in vivo MRE, n = 10. On the right, mean and SD are given for distances from peak fluorescence intensity (in μm) from ex vivo AFM, 0 μm n = 15, 100 μm n = 15, 200 μm n = 15, 300 μm n = 10, 400 μm n = 5.

## Correlation of ex vivo AFM with GFP-expression under the nestin promoter

**Figure 5:**
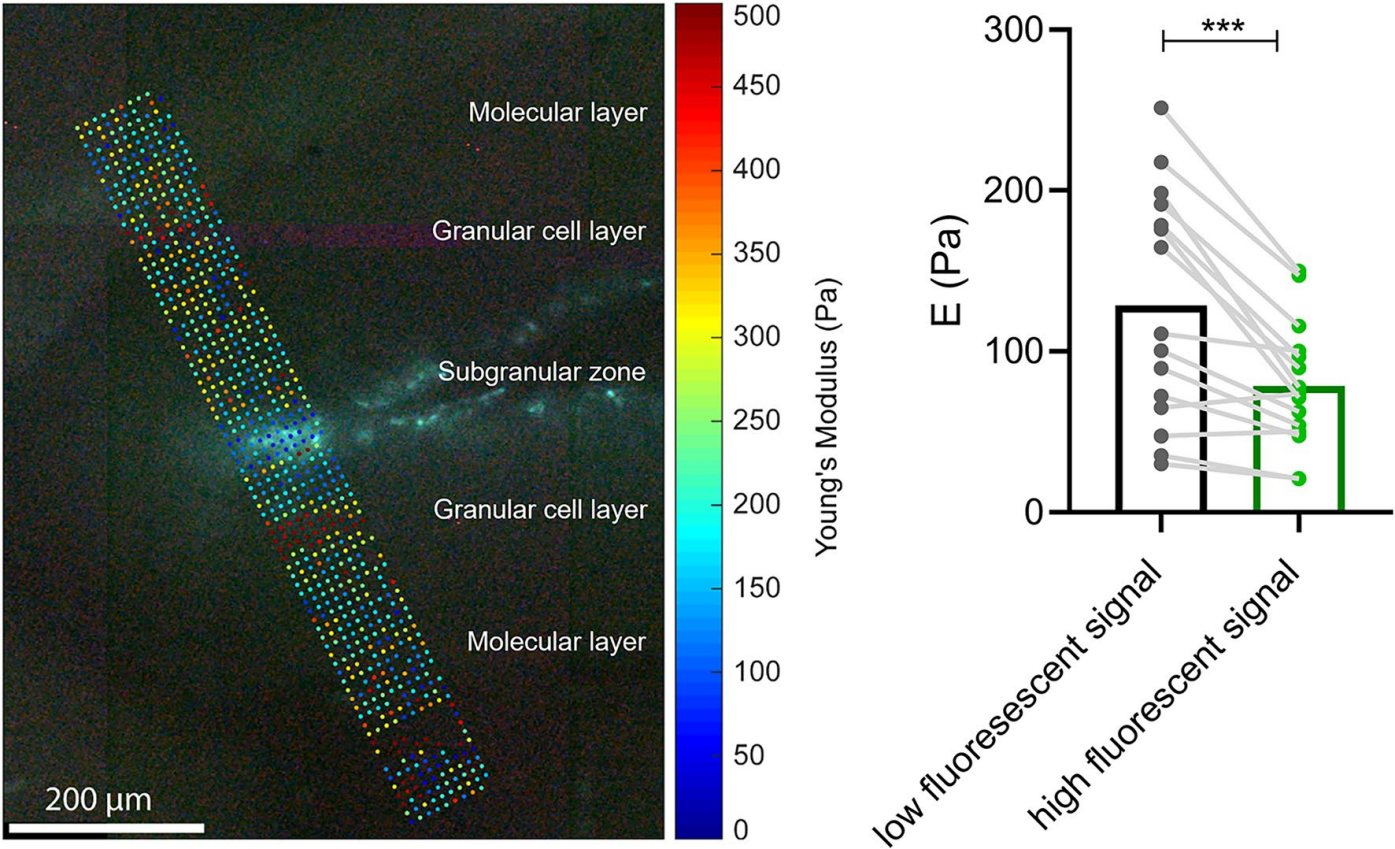
Ex vivo AFM results. On the left, a representative measurement profile overlaid on the high fluorescence region is shown (more data are shown in Supplementary Fig. 2). Right, the subgranular zone (area with high fluorescent signal) is significantly softer than the surrounding tissue (low fluorescent signal), n = 15, ***p = 0.0004.

Neurogenic activity was quantified from the intensity of the GFP fluorescence signal, and a corresponding measurement profile was placed on the SGZ as shown Fig. 5 (left).

**Figure 6:**
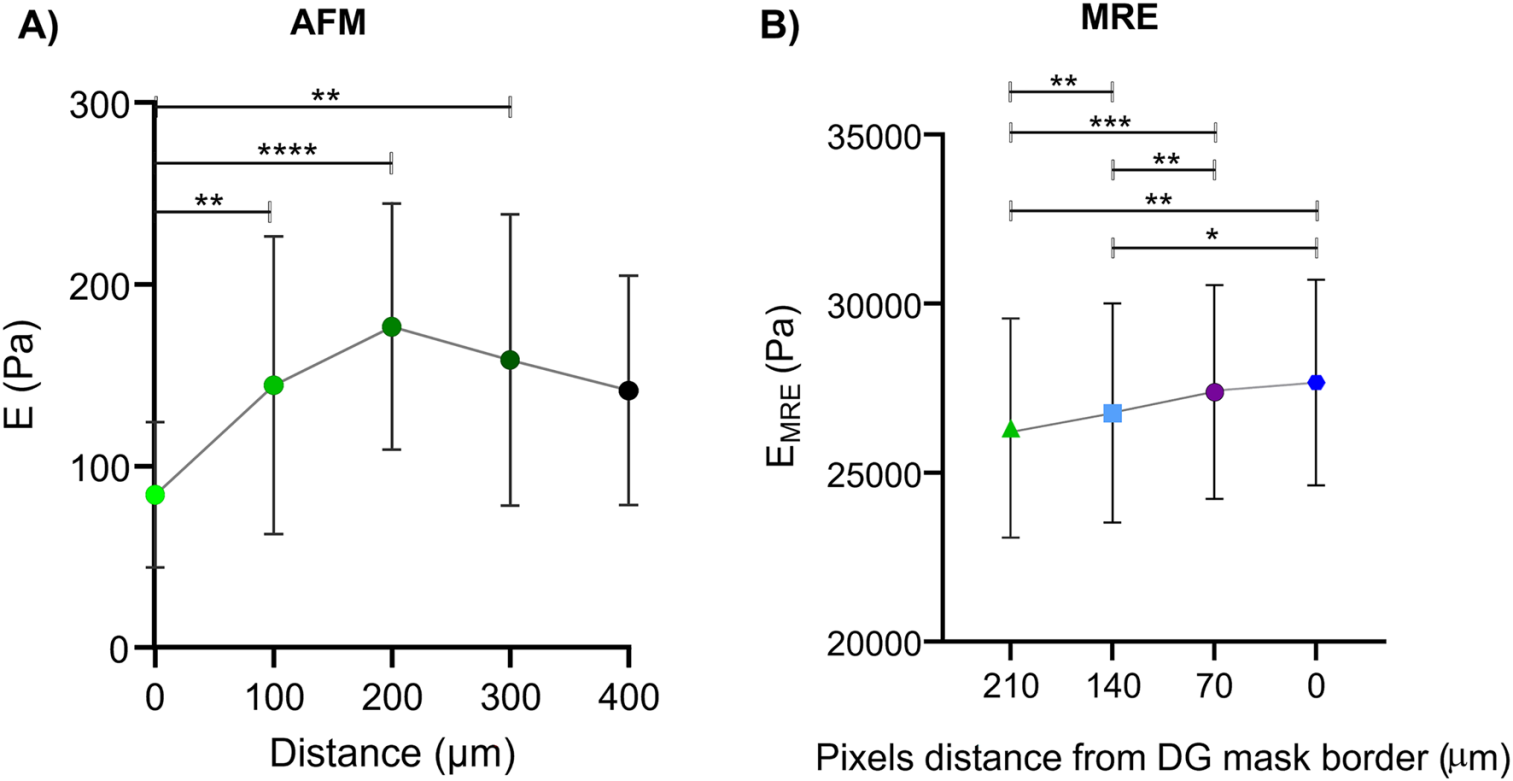
Comparison of ex vivo AFM and in vivo MRE results. (**A**) Stiffness increases with greater distance (in µm) from the peak of the fluorescence signal (marked as 0), 0 µm vs. 100 µm **p = 0.0025; 0 µm vs. 200 µm ****p < 0.0001; 0 µm vs. 300 µm **p = 0.0069, 0 µm n = 15, 100 µm n = 15, 200 µm n = 15, 300 µm n = 10, 400 µm n = 5. (**B**) Similar results are seen in vivo when the dentate gyrus mask is eroded: original mask (0 µm) vs. 2 pixel eroded mask (140 µm), *p = 0.0309, eroded original mask (0 µm) vs. 3 pixels eroded mask (210 µm), **p = 0.0043; 1 pixel eroded (70 µm) vs. 2 pixel eroded mask (140 µm), **p = 0.0051; 1 pixel eroded (70 µm) vs. 3 pixel eroded mask (210 µm), ***p = 0.0005; 2 pixel eroded (140 µm) vs. 3 pixel eroded mask (210 µm), **p = 0.0069), n = 10.

The 15 AFM measurements shown in Fig. 5 (right) reveal that Young’s modulus E is lower in areas of high fluorescence signal (covering the SGZ) than in areas of low GFP intensity (128 ± 72 Pa *vs*. 78 ± 39 Pa, p = 0.0004, Fig. 5). Furthermore, as shown in Fig. 6, E (Pa) significantly increases with increasing distance from peak fluorescence (SGZ). As can be seen in Fig. 2, the fluorescence signals were not spatially associated with distinct changes in cell nuclear density (DAPI), suggesting that the observed softening within the SGZ was not related to the density of cell nuclei.

Absolute values are compiled in Table 1. Relative changes in E (Pa) with distance from the peak signal (SGZ) are: 71.9% at 100 µm (p = 0.0025), 110.2% at 200 µm (p < 0.0001), and 88.4% at 300 µm (p < 0.0069) while no significant change is seen at 400 µm (p = 0.0699). Figure 6 also shows MRE results for E_MRE_, indicating consistent softening towards SGZ in vivo (significances are given in Fig. 6 and absolute values in Table 1).

## Discussion

In this study, we compared AFM and MRE to investigate whether the viscoelastic properties of the murine DG in vivo reflect similar patterns of stiffness reduction as observed ex vivo in correlation with neurogenic function. In vivo MRE results show that the DG is softer than AH with markedly soft properties towards the SGZ region. Using AFM, we were able to (1) confirm the markedly soft properties of the SGZ and (2) correlate this property with GFP-labeled nestin signal, indicating the functional importance of this viscoelastic signature.

It has been shown before that MRE is sensitive to microscopic mechanical interactions such as collagen crosslinking on the nanoscale^37^, tumor cell stiffness on the micrometer scale^38^, and wave scattering on the submillimeter scale^39^. Our study now provides additional evidence about the mechanical signature of microscopic regions involved in adult neurogenesis to which in vivo MRE is potentially sensitive. The mechanical properties of these regions are highly relevant in brain pathophysiology, as demonstrated by numerous studies using in vivo elastography or ex vivo indentation methods such as AFM^29,30,40–45^.

Using in vivo MRE we here show mechanical heterogeneity of the murine hippocampus between the Ammons’s horn and the dentate gyrus. Consistent with our result, recent studies on human hippocampal subfields showed significant differences in stiffness^18^ and damping ratio^18,24^ between the dentate gyrus combined with the CA3 region and the CA1/2 subfields of the Ammon´s horn. This mechanical heterogeneity of the hippocampus, as found here by noninvasive in vivo MRE, is in agreement with previous ex vivo AFM indentation measurements showing that the DG of adult rats has softer elastic properties than the subregions of the AH^46^. Moreover, the rodent DG with its subregions has been shown to be heterogeneous in terms of stiffness as revealed by ex vivo indentation measurement^16,47^. In these studies, the granular cell layer was found to be softer than surrounding regions such as the hilus and the molecular layer^16,47^. Softening correlated negatively with neuronal and nuclei density^16,47^ and positively with the presence of astrocytes^47^. However, these studies did not specifically examine the SGZ and most likely included the SGZ in the granular cell layer^16,47^, making these results consistent with our findings of gradual softening towards the SGZ both ex vivo and in vivo. When the SGZ was examined individually by ex vivo indentation measurement and compared directly with its surrounding structures, it also exhibited softer mechanical properties^15^, which is consistent with our findings.

The relevance of tissue mechanical properties for neurogenic function is still under investigation. Other AFM studies in the subependymal zone, which is the largest neuronal stem cell niche in the rodent brain, suggest the importance of a relative stiffness gradient that directs migratory behavior and differentiation of newborn neurons^48^. Neuroblasts generated in the subependymal zone migrate via the rostral migratory stream to the olfactory bulb, where they differentiate and integrate into existing networks^49^. Migratory cells that depart from the rostral migratory stream differentiate into granule cells or periglomerular cells. These cells reside in the granule cell layer and glomerular cell layer of the olfactory bulb^49^, which have been shown to be stiffer than the rostral migratory stream and the subependymal zone^48^, suggesting that durotaxis is critical for neuron migration. Therefore, as a neurogenic niche, the subependymal zone can be stiffer than the non-neurogenic parenchyma as long as it has softer properties than the target region^48^. Consistent with this, our results suggest that newly formed hippocampal neurons migrate^50^ from the softer SGZ into the stiffer granular cell layer of the DG.

Combined ex vivo and in vivo stiffness measurements of the murine hippocampus using invasive in vivo ultrasound-based shear-wave elasticity imaging and ex vivo AFM measurements have been performed before^51^. However, in this study, the SGZ was not analyzed separately but in conjunction with the hilus ex vivo while in vivo stiffness was measured for the whole hippocampus^51^*.* To our knowledge, no other study has compared the viscoelastic properties of the intact brain measured noninvasively with the spatial representation of neurogenic function. The unique combination of techniques, GFP-labeled nestin-positive cells, AFM, and MRE allowed us to match tissue stiffness in within the DG with nestin expression.

Although our results are encouraging, our study has limitations. First, direct comparison of ex vivo AFM and in vivo MRE is naturally hampered by the lacking registration of AFM images with MRE because AFM covers only small areas of the image slices obtained by MRE. Therefore, the location of the SGZ in MRE parameter maps had to be estimated, which was further impeded as standardized atlas masks of the SGZ are not yet available. In addition, we cannot fully rule out that MRE measurements were influenced by partial volume effects induced by the ventricles in the vicinity of the measured DG. However, erosion of the DG masks resulted in progressively smaller masks centered in the SGZ area and moving increasingly farther away from the boundaries. Therefore, an increase rather than a decrease in values with distance from the DG boundaries would be expected as a result of partial volume effects. Furthermore, previous MRE studies have studied regions of comparable size and border proximity to DG as analyzed here^28,29^. Possible limitations of our AFM experiments relate to sample transfer in a free-floating 24-well plate and brief contact with a clean glass surface during immobilization. In addition, absolute E-values may have been affected by the stiffness of the cantilever, the size of the beads, and indentation velocities. Nevertheless, we carefully adjusted these parameters to ensure that they were within the limits where the linear Hertz model can still be safely applied. Simulations have shown that, for spherical tips, the strain field extends about five times the penetration depth into the sample and that the penetration depth should not exceed 1/3 of the probe diameter^52^. We therefore assume that AFM measured the effective mechanical properties beneath the contact area of approximately 8 µm^2^ including the first two cell layers with microenvironment. Given the consistency of our results across different samples and measurement positions, we consider our AFM data to be a robust marker of the effective stiffness beneath the cantilever tip as sensed by nestin-expressing cells. The discrepancy between the quantitative values of in vivo MRE and ex vivo AFM may be attributed to two main technical differences: First, different length scales (8 µm^2^ in AFM versus 32,400 µm^2^ in-plane pixel size in MRE), and, second, different dynamic ranges (quasi-static AFM versus 1–1.4 kHz MRE vibrations). While AFM values are still in the order of magnitude of those measured with MRE at low frequencies^53^, MRE stiffness increases significantly from low to high frequencies due to viscoelastic dispersion^54^. Thus, the dynamic range appears to be more relevant than the scale difference, raising the prospect of using viscoelastic models in the future to match AFM and MRE over the broad dynamic range covered by both modalities.

Using ex vivo AFM, we showed here that tissue softening correlates with neurogenic signal intensity in the SGZ, and that softer properties can be found by in vivo MRE when the same region is analyzed, but at a much coarser scale. Both, in vivo MRE and ex vivo AFM, revealed consistent mechanical properties of the SGZ compared with surrounding regions. Collectively, our results contribute to the understanding of how viscoelastic tissue properties critically shape the biophysical environment that promotes neuronal proliferation, homeostasis, and repair. In the future, our results may be leveraged for neuronal regenerative medicine.

## Supplementary Information

Supplementary Information.

## Data Availability

The datasets generated during and analyzed during the current study are available from the corresponding author on reasonable request.

## Abbreviations

aCSF: Artificial cerebrospinal fluid
AFM: Atomic force microscopy
AH: Ammon’s horn
DG: Dentate gyrus
ECM: Extracellular matrix
FOV: Field of view
min: Minutes
mM: Millimols
mm: Millimeters
ms: Milliseconds
MRE: Magnetic resonance elastography
MRI: Magnetic resonance imaging
Pa: Pascal
PBS: Phosphate-buffered saline
PFA: Paraformaldehyde
RARE: Rapid acquisition with relaxation enhancement
RT: Room temperature
SD: Standard deviation
SGZ: Subgranluar zone
TA: Acquisition time
TE: Echo time
TR: Repetition time
