# Supplementary material for: Mechanical properties of murine hippocampal subregions investigated by atomic force microscopy and in vivo magnetic resonance elastography
Source: Sci Rep. 2022 Oct 6;12:16723. doi: 10.1038/s41598-022-21105-7 (PMC9537158; doi:10.1038/s41598-022-21105-7)
Supplement: Supplementary file 1 — Supplementary Information. [file 41598_2022_21105_MOESM1_ESM.pdf]

Supplementary Information to

Mechanical properties of murine hippocampal subregions investigated by atomic force microscopy and *in vivo* magnetic resonance elastography

Anna S. Morr[1], Marcin Nowicki, Gergely Bertalan[1], Rafaela Vieira Silva[3,4], Carmen Infante Duarte[3], Stefan Paul Koch[5,6], Philipp Boehm-Sturm[5,6], Ute Krügel[7], Jürgen Braun[8], Barbara Steiner[9], Josef A. Käs[10], Thomas Fuhs[10], Ingolf Sack*[1]

1. Charité - Universitätsmedizin Berlin, Corporate Member of Freie Universität Berlin and Humboldt-Universität zu Berlin, Department of Radiology, Berlin, Germany
2. Institute of Anatomy, University of Leipzig, Leipzig, Germany
3. Experimental and Clinical Research Center, Max Delbrück Center for Molecular Medicine in the Helmholtz Association (MDC) and Charité - Universitätsmedizin Berlin, Corporate Member of Freie Universität Berlin and Humboldt-Universität zu Berlin
4. Charité - Universitätsmedizin Berlin, Einstein Center for Neurosciences Berlin, Berlin, Germany
5. Charité - Universitätsmedizin Berlin, Corporate Member of Freie Universität Berlin and Humboldt-Universität zu Berlin, Department of Experimental Neurology and Center for Stroke Research, Berlin, Germany
6. Charité - Universitätsmedizin Berlin, NeuroCure Cluster of Excellence and Charité Core Facility 7T Experimental MRIs, Berlin, Germany
7. Rudolf Boehm Institute of Pharmacology and Toxicology, University of Leipzig, Leipzig, Germany
8. Charité – Universitätsmedizin Berlin, Corporate Member of Freie Universität Berlin and Humboldt - Universität zu Berlin, Institute of Medical Informatics, Berlin, Germany
9. Charité – Universitätsmedizin Berlin, Corporate Member of Freie Universität Berlin and Humboldt - Universität zu Berlin, Clinic for Neurology and Experimental Neurology, Berlin, Germany
10. Section of Soft Matter Physics, Peter Debye Institute for Soft Matter Physics, Faculty of Physics and Geosciences, University of Leipzig, Leipzig, Germany

***Corresponding Author**

Ingolf Sack, PhD

Department of Radiology

Charité – Universitätsmedizin Berlin

Charitéplatz 1, 10117 Berlin, Germany

ingolf.sack@charite.de

Supplementary Figure 1

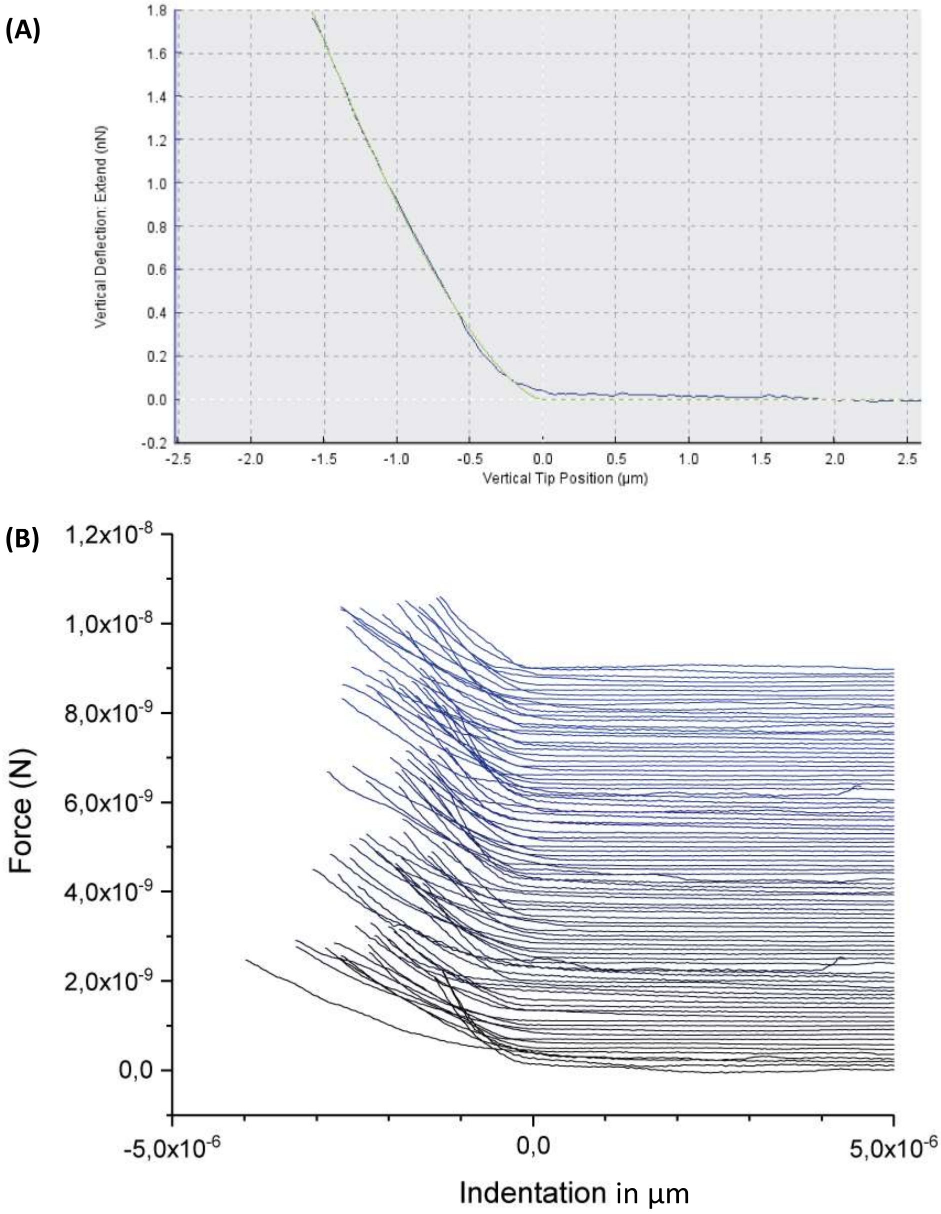

Supplementary Figure 1 Force indentation curves. **(A)** AFM acquires force-distance curves (blue curve), which are then shifted such that the contact point is at the origin. A simple Hertz model according to equation (1), main text, is fitted (green curve) to each measurement curve to recover Young's modulus, which basically determines the slope of the measurement curve. **(B)** A series of approximately 80 measurement curves showing smooth curvatures and indicating the validity of the Hertz model with different Young's moduli (slopes).

Supplementary Figure 2

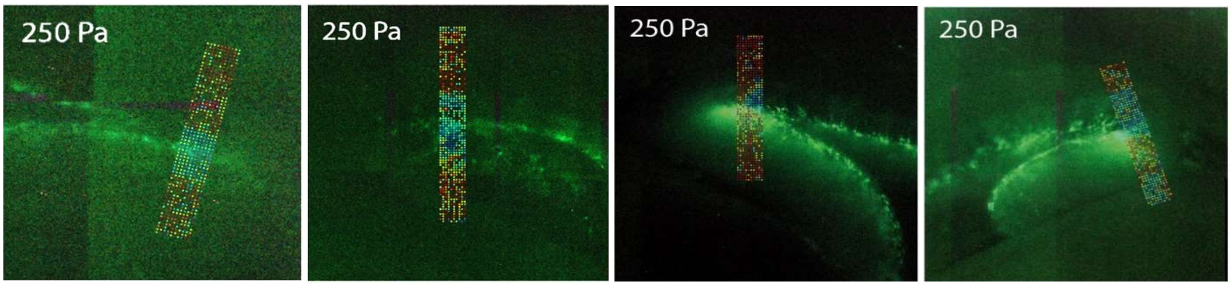

Supplementary Figure 2 Fluorescent image overlayed with Young's modulus maps from atomic force microscopy (AFM). Green color indicates nestin expressing cells as a marker of neurogenic activity. The color scale of the Young's modulus heat maps runs from 0 to 250 Pa as indicated in the images.
